# Transcriptional profiling of Chinese medicinal formula Si-Wu-Tang on breast cancer cells reveals phytoestrogenic activity

**DOI:** 10.1186/1472-6882-13-11

**Published:** 2013-01-10

**Authors:** Mandy Liu, Jeffery Fan, Steven Wang, Zhijun Wang, Charles Wang, Zhong Zuo, Moses SS Chow, Leming Shi, Zhining Wen, Ying Huang

**Affiliations:** 1 Department of Pharmaceutical Sciences and Center for Advancement of Drug Research, College of Pharmacy, Western University of Health Sciences, Pomona, California; 2 Functional Genomics Core, Beckman Research Institute, City of Hope Comprehensive Cancer Center, Duarte, California; 3 School of Pharmacy, Faculty of Medicine, The Chinese University of Hong Kong, Shatin, New Territories, Hong Kong, China; 4 National Center for Toxicological Research, US Food and Drug Administration, Jefferson, Arkansas; 5 Department of Clinical Pharmacy and Center for Pharmacogenomics, School of Pharmacy, Fudan University, Shanghai, China; 6 College of Chemistry, Sichuan University, Chengdu, Sichuan, China

**Keywords:** Phytoestrogens, Microarrays, Genomics, Chemoprevention, Breast cancer, Herbal medicines, Transcriptional profiling

## Abstract

**Background:**

Si-Wu-Tang (SWT), comprising the combination of four herbs, Paeoniae, Angelicae, Chuanxiong and Rehmanniae, is one of the most popular traditional oriental medicines for women’s diseases. In our previous study, the microarray gene expression profiles of SWT on breast cancer cell line MCF-7 were found similar to the effect of β-estradiol (E2) on MCF-7 cells in the Connectivity Map database.

**Methods:**

Further data analysis was conducted to find the main similarities and differences between the effects of SWT and E2 on MCF-7 gene expression. The cell proliferation assay on MCF-7 (ER-positive) and MDA-MB-231 (ER-negative) cells were used to examine such estrogenic activity. The estrogenic potency of SWT was further confirmed by estrogen-responsive element (ERE) luciferase reporter assay in MCF-7 cells.

**Results:**

Many estrogen regulated genes strongly up-regulated by E2 were similarly up-regulated by SWT, e.g., *GREB1*, *PGR* and *EGR3*. Of interest with regard to safety of SWT, the oncogenes *MYBL1* and *RET* were strongly induced by E2 but not by SWT. Quantitative RT-PCR analysis revealed a highly concordant expression change in selected genes with data obtained by microarrays. Further supporting SWT’s estrogenic activity, in MCF-7 but not in MDA-MB-231 cells, SWT stimulated cell growth at lower concentrations (< 3.0 mg/ml), while at high concentrations, it inhibits the growth of both cell lines. The growth inhibitory potency of SWT was significantly higher in MDA-MB-231 than in MCF-7 cells. The SWT-induced cell growth of MCF-7 could be blocked by addition of the estrogen receptor antagonist tamoxifen. In addition, SWT was able to activate the ERE activity at lower concentrations. The herbal components Angelicae, Chuanxiong and Rehmanniae at lower concentrations (< 3.0 mg/ml) also showed growth-inducing and ERE-activating activity in MCF-7 cells.

**Conclusions:**

These results revealed a new mechanism to support the clinical use of SWT for estrogen related diseases and possibly for cancer prevention. This study also demonstrated the feasibility of using microarray transcriptional profiling to discover phytoestrogenic components that are present in natural products.

## Background

Oriental medicinal herbs provide a promising source to develop orally effective, non-toxic, complementary and alternative medicine (CAM) modality for cancer prevention. Si-Wu-Tang [SWT, Si-Wu decoction (Chinese name), Samultang (Korean name), or Shimotsu-to (Japanese name)], comprising the combination of four herbs, Paeoniae, Angelicae, Chuanxiong and Rehmanniae, is one of the most popular traditional oriental medicines for women’s health [1]. It has been used in Eastern Asia for about one thousand years for various women’s diseases and ranks first as the most frequently used Chinese medicines in several surveys [2]. It is an inexpensive over-the-counter preparation used for the relief of menstrual discomfort, climacteric syndrome, peri- or postmenopausal syndromes and other estrogen-related diseases [1-5]. The major principle of SWT therapy as Chinese Medicine is to improve a deficiency of Qi and Blood [6]. SWT has shown sedative, anti-coagulant and anti-bacterial activities as well as protective effect on radiation-induced bone marrow damage in model animals [7,8]. Several *in vitro* and *in vivo* studies show a preventive effect of SWT on endometrial carcinogenesis induced by carcinogen and estrogen [9,10], although the mechanisms and active constituents are unknown. In a pilot clinical trial on the effects of SWT in the treatment of primary dysmenorrhoea, the administration of SWT was well tolerated without any adverse reactions [2]. Another clinical study demonstrated that SWT can be integrated as an alternative therapy within Western medicine [11].

Despite the wide use of SWT for women’s diseases, little is known for its potential estrogenic properties. In our previous study [12], the microarray gene expression profiles of SWT on human breast cancer cell line MCF-7 were compared with 1,309 compounds in the “Connectivity Map” (cMAP) reference database [13]. The profile of SWT-treated MCF-7 cells showed the highest match with that of estradiol (E2)-treated MCF-7 cells in the cMAP database [12], consistent with SWT’s widely claimed use for women’s diseases and suggesting an estrogen-like effect. Such results indicate that SWT may contain phytoestrogen(s), which are a diverse group of plant-derived compounds that structurally or functionally mimic endogenous estrogens [14]. Many lines of evidence suggested that phytoestrogens not only may be useful as an alternative and complementary approach for hormone replacement therapy, but also for the prevention of breast or prostate cancers [15,16]. Studies on phytoestrogens over the past few decades has greatly increased, although these research results indicate both health benefit and risk for the application of phytoestrogens [17]. A recent survey reported that almost 30% of women sought CAM therapies such as soy or other herbal products, to combat postmenopausal discomfort [18]. As the number of women who seek the use of herbal medicinal products is increasing, new methods are required to evaluate the efficacy and adverse reactions of phytoestrogen components.

It has been previously reported that phytoestrogens and the natural estrogens such as E2 can induce a similar effect on gene expression profiles of a panel of “estrogen-responsive genes” [19]. DNA microarray – based expression profiling has been used as a genomic approach for the characterization of compounds with estrogen-like activities. For examples, a customized DNA microarray containing 172 estrogen-responsive genes have been used to evaluate the effect of multiple well known phytoestrogens including genistein and daidzein [19], and the industrial endocrine disruptors including zearalenone, diethylstilbestrol and dioxin [20-23]. These results obtained using DNA microarrays were consistent with those derived from other bioassays that are used for detecting estrogenic activity, such as ligand-binding and reporter gene assays. However, this genomic approach has not yet been applied to herbal products used in oriental medicines. Moreover, including only selected gene sets in customized DNA microarray may result in a bias in gene selection. Therefore, we hypothesize that the whole genome expression analysis based on available microarray datasets can provide a comprehensive and unbiased approach to identifying new phytoestrogens from natural products or dietary components, revealing novel mechanisms, and/or providing a quality control for the evaluation of natural products with phytoestrogen components.

The purpose of the present study is to examine the phytoestrogenic effect of SWT using the whole human genome microarray analysis followed by pharmacological studies. We firstly re-analyzed the microarray gene expression data to find the similarities and differences between the effect of SWT and E2 on gene expression of MCF-7 cells. Real-time RT-PCR analysis was used to validate the microarray data. Cell growth and estrogen receptor assays were used to confirm the findings from genomic analysis. This study provides insights in understanding the complex actions of SWT as a potential estrogen receptor modulator and scientific evidence to support the empirical clinical use of SWT.

## Methods

### Compounds

17 β-estradiol, tamoxifen, 4-OH tamoxifen and DMSO were purchased from Sigma-Aldrich (St. Louis, MO, USA).

### Preparation of SWT extracts

The SWT products and its four single herb extracts were obtained from the School of Pharmacy, Chinese University of Hong Kong. These products were manufactured under GMP condition at the Hong Kong Institute of Biotechnology (Hong Kong, China) according to the protocol described in Chinese Pharmacopoeia 2005 [24] with modifications. The standard adult dosage of SWT extracts is 15 grams per day [11]. Crude water extracts were prepared from powdered SWT. Fresh extracts were prepared right before the experiment. The extract was prepared by dissolving the powder into PBS buffer or culture medium, followed by sonication for 30 min.

### Cell lines and cell culture

The MCF-7 cells were purchased from American Type Culture Collection (ATCC, Manassas, VA, USA), cultured in Dulbecco’s modified Eagle’s medium (DMEM) supplemented with 10% fetal bovine serum (FBS), 1% non-essential amino acids, 100 unit/mL penicillin, 100 μg/mL streptomycin, 1 mM sodium pyruvate, and 2 mM L-glutamine in an atmosphere of 5% CO_2_ at 37°C. For microarray analysis, the cells were seeded in 6-well plates at a density of 1 × 10^5^ cells/ml. After incubating for 24 hours and at least 4 days before treatment, the medium was then replaced by hormone free medium which contains phenol-red free DMEM medium supplemented with 5% charcoal-dextrin stripped FBS (CD-FBS) to prevent the influence of hormones or estrogen-like compounds in the regular culture medium. The MCF-7 cells were then incubated with hormone free medium and treated by 0.001% DMSO (vehicle control group, C), 0.1 μM 17 β-estradiol (EM), 0.0256, 0.256, and 2.56 mg/ml SWT (SL, SM and SH) for 6 hours. The concentrations of SWT were determined based on previous *in vitro* studies [25]. Three replicates for each of the five treatment groups were analyzed. The detailed experimental information including names and concentrations of the treatments are shown in previous report [12].

### RNA extraction and microarray processing

Total RNA was extracted using RNeasy Mini Kit (QIAGEN, Valencia, California), following the manufacturer’s protocol. The concentrations of RNA were measured by a NanoVue Plus (GE Healthcare, Piscataway, NJ, USA) and adjusted to 0.2 μg/μl. The RNA samples were stored at −80°C before further processing for microarray analysis or cDNA synthesis. Method of microarray processing is reported [12].

### Microarray data analysis

Microarray data specifically generated for this study are MIAME compliant. The raw data are available through the National Center for Biotechnology Information’s Gene Expression Omnibus (GEO series accession number: GSE23610). The microarray gene expression data were imported to ArrayTrack [26], a software system developed by the U.S. Food and Drug Administration’s National Center for Toxicological Research for the management, analysis, visualization and interpretation of microarray data (http://www.fda.gov/ArrayTrack/). The software of CLUSTER and

TREEVIEW were used to cluster and visualize the data by using the correlation metric and average linkage [27]. For each probeset, log2-transformed intensity data were used in a two-sample *t*-test to obtain a *p* value and a fold change (FC).

### Real-time RT-PCR

To validate the microarray results, one microgram of total RNA was incubated with DNase I, and reverse transcribed with oligo dT using Superscript II RT-PCR (Invitrogen). One microliter of RT product was amplified by primer pairs specific for *GREB1*, *PGR*, *MYBL1*, *RET* and *ST8SIA4*. The *GAPDH* gene was used as a normalizing control. The primer sequences are *GREB1*: 5^′^-ATCCTGAACGTGGACCTGAC-3^′^ and 5^′^-CACCACGATCTGCTTCTTCA-3^′^; *MYBL1*: 5^′^-GAAAAATGCGAGTGGGTCAT-3^′^ and 5^′^-CCCACAAATAGGGGTTGATG-3^′^; *PGR*: 5^′^-AAATCATTGCCAGGTTTTCG-3^′^ and 5^′^-TGCCACATGGTAAGGCATAA-3^′^; *RET*: 5^′^-ACAGGGGATGCAGTATCTGG-3^′^ and 5^′^-CCTGGCTCCTCTTCACGTAG-3^′^; *ST8SIA4*: 5^′^-CGAACTGCCTATCCGTCATT-3^′^ and 5^′^-CTTAGGGAAGGGCCAGAATC-3^′^; *GAPDH*: 5^′^-AGCCACATCGCTCAGACAC-3^′^ and 5^′^-GCCCAATACGACCAAATCC-3^′^. Relative gene expression was measured using the GeneAmp 7300 Sequence Detection system (Applied Biosystems, Foster City, CA, USA) using a SYBR Green protocol. For all amplifications, a standard amplification program was used (1 cycle of 50°C for 2 min, 1 cycle of 95°C for 10 min, 50 cycles of 95°C for 15 s and 60°C for 1 min). At the end of PCR cycling steps, data for each sample was displayed as a melting curve. The ABI SDS software was used to determine a “Cycle Threshold” (Ct), which was the cycle number where the linear phase for each sample crossed the threshold level. All samples were run in triplicate with no-template control.

### Cell proliferation assay

Growth-inhibitory activity on MCF-7 and MDA-MB-231 cells was tested using a proliferation assay with sulforhodamine B (SRB), a protein-binding reagent (Sigma), or MTS assay (Promega) as described before [28]. Both SRB and MTS assays showed consistent results and therefore were used interchangeably. 2000–5000 cells/well were seeded in 96-well plates and incubated for 24 h in RPMI-1640. The medium was then replaced by hormone free medium which contains phenol-red free DMEM medium supplemented with 5% CD-FBS 3 days before drug treatment. The cells were then incubated with hormone free medium and treated by test agents added in a dilution series in three or six replicate wells for incubation of days designated in Results. To determine IC_50_ values, the absorbance of control cultures without drug was set at 1. Dose–response curves were plotted using GraphPad Prism (San Diego, California). Each experiment was performed independently at least twice. Student’s *t* test was used to determine the degree of significance.

### Luciferase reporter gene assay

The MCF-7 cells were cultured in hormone-free medium for three days and then plated in 96-well plates. The luciferase reporter construct ERE-luc was a gift from Dr. David Sanchez at Western University of Health Sciences. The MCF-7 cells were transfected with the ERE-luc plasmid and a constitutively active renilla luciferase (pRL-TK-luc, from Promega; to correct for transfection efficiency) (10:1 ratio) using FuGENE HD Transfection Reagent (Roche Applied Science, Indianapolis, IN) according to the manufacturer’s instructions. 24 hours after transfection, the cells were exposed to the extracts of SWT or components, or E2 (10 nM), in the presence or absence of tamoxifen, for another 24 hours. Cell lysates were used for determining luciferase activities of both firefly and renilla by the dual luciferase reporter gene assay (Promega). Firefly luciferase activity was normalized to renilla luciferase activity. Each experiment was carried out in triplicate and expressed as the mean ± Standard Error (SE). Student’s *t* test was used to determine the degree of significance.

## Results

### Comparison of the expression changes induced by SWT and E2

**Figure 1:**
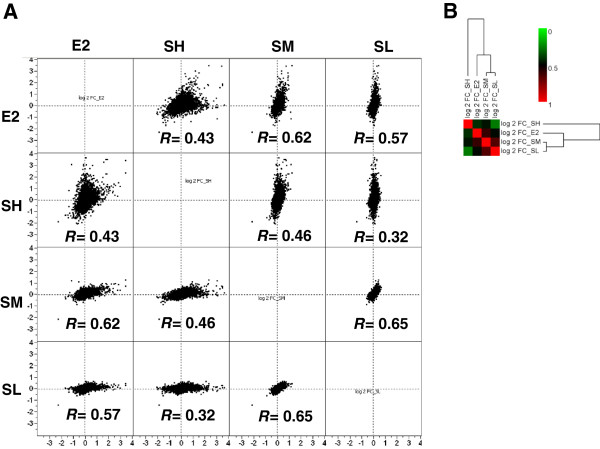
**Similarity between SWT and E2 treatment examined using whole genome DNA microarray.** (**A**) Scatterplot graph shown as the comparison of gene expression profiles between E2 and SWT treatment at various concentrations (SH, SM and SL). The axes show log2 FC calculated for each of the treatment condition (FC: fold change, derived from intensity for treatment/intensity for control). The Spearman correlation coefficient (*R*) between two profiles was calculated for each graph. *P* < 0.0001 for all the correlations. (**B**) The hierarchical clustering analysis and heatmap of the correlation coefficients of the gene expression profiles based on log2 FC for each treatment group. This genome-wide analysis shows that the low- and medium-concentration SWT treatments (SM and SL) showed higher similarity with E2 treatment, while the high concentration SWT (SH) induced a gene expression changes less similar with E2 treatment.

Spearman correlation analysis was applied to assess the overall similarity of the gene expression profiles between E2 and SWT in three concentrations (SH, SM and SL) using all the 54,675 probes on the microarrays. The correlations with E2 were significant for all the SWT concentrations in the order of SM (r = 0.62, *P* < 0.0001) > SL (r = 0.57, *P* < 0.0001) > SH (r = 0.43, *P* < 0.0001) (Figure 1A). The correlation was confirmed by hierarchical clustering analysis (Figure 1B). This genome-wide analysis indicates that gene expression of low- and medium-concentration SWT treatments (SM and SL) showed certain similarity with E2 treatment, while the high concentration SWT (SH) induced a gene expression changes more distinct with E2 treatment, possibly due to a dramatic treatment effect of high concentration of SWT.

The treatment by E2 resulted in large numbers of genes differentially expressed in MCF-7 cells in comparison with the vehicle controls. Applying the initial cutoff, *t*-test *P* value < 0.05 and fold change > 1.5, the treatment with E2 changed the expression of 830 unique genes (corresponding to 1,292 probe sets). We applied a more stringent selective filter to reduce the 1,292 E2-responsive probe sets to a 45-probe subset for further analysis (Table 1). The list includes genes that showed strongest up-regulation or down-regulations induced by E2 by applying a filtering cutoff, fold change > 4 for up-regulated genes, fold change < 0.4 for down-regulated genes, and false discovery rate (FDR) < 0.01. Since the same microarrays (Affymetrix Human Genome U133 Plus 2.0) have been used in several previous studies for the analysis of MCF-7 cells treated with E2 for 3, 6 or 12 hours [29-31], we compared the data derived from these studies available at the NCBI GEO Profiles at the site http://www.ncbi.nlm.nih.gov/geoprofiles. All of the 45 probes show the same trend of E2-induced up- or down-regulation for at least 2 folds in comparison with the controls. Therefore, although it is impossible for the selected 45 probes to include the whole set of genes regulated by the estrogen receptors, we believe that such selection represents a subset of reliable “estrogen-responsive genes” or “fingerprint of estrogen treatment” on the MCF-7 cells. Shown in Table 1, many genes strongly up-regulated by E2 were similarly up-regulated by SWT although to a lower degree, e.g., *C14orf182*, *PGR*, *RBM24*, *GREB1*, *RERG*, *SGK3*, all of which are well-known estrogen regulated genes. Of interest with regard to cancer prevention, the oncogenes *MYBL1*, *RET* and cyclin D1 (not listed in Table 1) is strongly induced by E2 but marginally by SWT (Table 1).

**Table 1:** Expression changes of estrogen-responsive genes in MCF-7 cells treated with E2 and SWT

			**Fold change (treatment/control)**		
**Affymetrix probe ID**	**Gene symbol**	**Description**	**E2**	**SH**	**SM**
***Up-regulated genes***					
237460_x_at	C14orf182	chromosome 14 open reading frame 182	**13.77**	**6.29**	1.18
228554_at	PGR	progesterone receptor	**12.10**	**2.83**	1.72
205862_at	GREB1	growth regulation by estrogen in breast cancer 1	**10.87**	**5.08**	2.37
206115_at	EGR3	early growth response 3	**10.69**	**10.70**	1.65
235004_at	RBM24	RNA binding motif protein 24	**10.23**	**6.07**	1.57
1557277_a_at			**8.71**	2.08	1.32
231120_x_at	PKIB	protein kinase (cAMP-dependent, catalytic) inhibitor beta	**7.96**	2.29	1.27
219525_at	SLC47A1	solute carrier family 47, member 1	**7.93**	2.22	1.40
205440_s_at	NPY1R	neuropeptide Y receptor Y1	**7.08**	**2.81**	1.84
44790_s_at	C13orf18	chromosome 13 open reading frame 18	**7.08**	**2.97**	2.59
213906_at	MYBL1	v-myb myeloblastosis viral oncogene homolog (avian)-like 1	**6.78**	1.26	1.29
220038_at	SGK3	serum/glucocorticoid regulated kinase family, member 3	**6.63**	**1.79**	1.51
222921_s_at	HEY2	hairy/enhancer-of-split related with YRPW motif 2	**6.11**	**2.88**	1.59
228241_at	AGR3	anterior gradient homolog 3 (Xenopus laevis)	**6.08**	**2.55**	1.84
204798_at	MYB	v-myb myeloblastosis viral oncogene homolog (avian)	**5.96**	**2.37**	1.92
227627_at	SGK3	serum/glucocorticoid regulated kinase family, member 3	**5.83**	**1.69**	1.54
208018_s_at	HCK	hemopoietic cell kinase	**5.77**	1.33	1.02
239777_at	C14orf182	chromosome 14 open reading frame 182	**5.63**	**3.29**	1.27
207886_s_at	CALCR	calcitonin receptor	**5.38**	**2.59**	1.86
232306_at	CDH26	cadherin 26	**5.37**	**3.51**	1.94
219743_at	HEY2	hairy/enhancer-of-split related with YRPW motif 2	**5.30**	**1.83**	1.37
208305_at	PGR	progesterone receptor	**5.22**	1.69	1.10
223551_at	PKIB	protein kinase (cAMP-dependent, catalytic) inhibitor beta	**5.18**	**2.13**	1.42
211421_s_at	RET	ret proto-oncogene	**4.98**	**1.54**	1.19
205326_at	RAMP3	receptor (G protein-coupled) activity modifying protein 3	**4.67**	**2.35**	1.87
244745_at	RERG	RAS-like, estrogen-regulated, growth inhibitor	**4.61**	1.43	1.51
237334_at			**4.55**	2.25	1.34
219702_at	PLAC1	placenta-specific 1	**4.45**	2.02	1.09
223201_s_at	TMEM164	transmembrane protein 164	**4.44**	**1.68**	1.23
219825_at	CYP26B1	cytochrome P450, family 26, subfamily B, polypeptide 1	**4.42**	1.26	1.19
224428_s_at	CDCA7	cell division cycle associated 7	**4.39**	1.11	1.45
209687_at	CXCL12	chemokine (C-X-C motif) ligand 12	**4.31**	1.77	1.29
242064_at	SDK2	sidekick homolog 2 (chicken)	**4.25**	1.48	0.99
219985_at	HS3ST3A1	heparan sulfate (glucosamine) 3-O-sulfotransferase 3A1	**4.17**	2.01	1.40
220486_x_at	TMEM164	transmembrane protein 164	**4.09**	1.64	1.22
201739_at	SGK1	serum/glucocorticoid regulated kinase 1	**4.01**	**1.86**	1.29
**Down-regulated genes**					
242943_at	ST8SIA4	ST8 alpha-N-acetyl-neuraminide alpha-2,8-sialyltransferase 4	**0.31**	**0.40**	0.85
202948_at	IL1R1	interleukin 1 receptor, type I	**0.32**	**0.40**	0.78
229354_at	AHRR	aryl-hydrocarbon receptor repressor	**0.33**	**0.83**	0.80
230261_at	ST8SIA4	ST8 alpha-N-acetyl-neuraminide alpha-2,8-sialyltransferase 4	**0.36**	**0.41**	0.88
230836_at	ST8SIA4	ST8 alpha-N-acetyl-neuraminide alpha-2,8-sialyltransferase 4	**0.37**	**0.35**	0.88
228176_at	EDG3	endothelial differentiation, sphingolipid G-protein-coupled receptor, 3	**0.38**	0.67	0.95
205749_at	CYP1A1	cytochrome P450, family 1, subfamily A, polypeptide 1	**0.38**	**1.76**	0.97
213413_at	BG434174	STON1	**0.39**	**0.27**	0.63

Gene expression of MCF-7 cells after 6 hours of treatment with 0.1 μM E2 or SWT (2.56, 0.256 or 0.0256 mg/ml) was analyzed by microarrays. Listed are selected genes that showed strongest up-regulation or down-regulations induced by E2 by applying the cutoff of fold change > 4 for up-regulated genes, fold change < 0.4 for down-regulated genes, and false discovery rate (FDR) < 0.01. Expression values are shown by fold changes between treatment and control, i.e., expression values relative to untreated control cells for E2, SH and SM. Fold changes in bold: FDR < 0.01.

**Figure 2:**
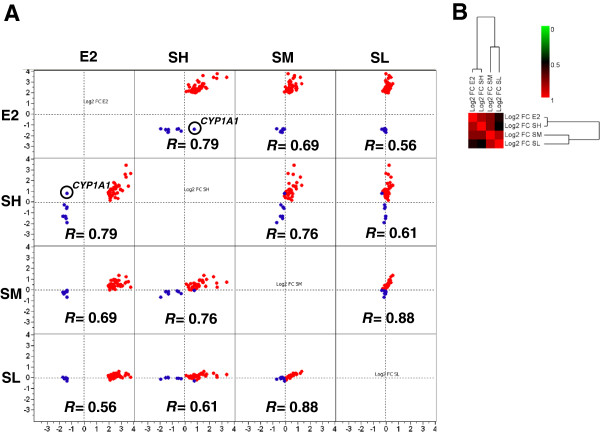
**Similarity between SWT and E2 treatment on “estrogen-responsive genes”. (A**) Scatterplot graph shown as the comparison of gene expression profiles of 45 selected probes between E2 and SWT treatment at various concentrations (SH, SM and SL). The axes show log2 FC calculated for each of the treatment condition. The Spearman correlation coefficient (*R*) between two profiles was calculated for each graph. The E2-up-regulated probes are red; E2-down-regulated probes are blue. *CYP1A1* on the plots is highlighted because it is down-regulated by E2 (blue) but up-regulated by SWT. *P* < 0.0001 for all the correlations. (**B**) The hierarchical clustering analysis and heatmap of the correlation coefficients between the gene expression profiles based on log2 FC for each treatment group. A high correlation coefficient, colored in red in the heatmap, means that the gene expression profiles from two microarrays are very similar. The analysis focusing on 45 estrogen-responsive genes shows that all the SWT treatment concentrations showed higher similarity with E2 treatment.

Spearman correlation analysis was also applied to assess the similarity of the gene expression profiles between E2, SH, SM and SL using the selected 45 probes on the microarrays, named as “estrogen-responsive genes”. By focusing on the small subset of genes highly related to E2 regulation, the correlation between E2 and SWT treatments was greatly increased. In particular, the correlation between E2 and SH increased to be the highest among all the concentrations of SWT tested. The correlations were significant for all the SWT concentrations in the order of SH (r = 0.79, *P* < 0.0001) > SM (r = 0.69, *P* < 0.0001) > SL (r = 0.56, *P* < 0.0001) (Figure 2A). The correlation was similarly confirmed by hierarchical clustering analysis (Figure 2B). A high correlation coefficient (colored in red in the heatmap of Figure 2B) means that the gene expression profiles from two microarrays are very similar. This result indicates that by filtering out gene expression changes induced by high concentration SWT which is not related to the phytoestrogenic activity, the SWT treatments at all concentrations showed a high degree of similarity as the E2 treatment.

**Figure 3:**
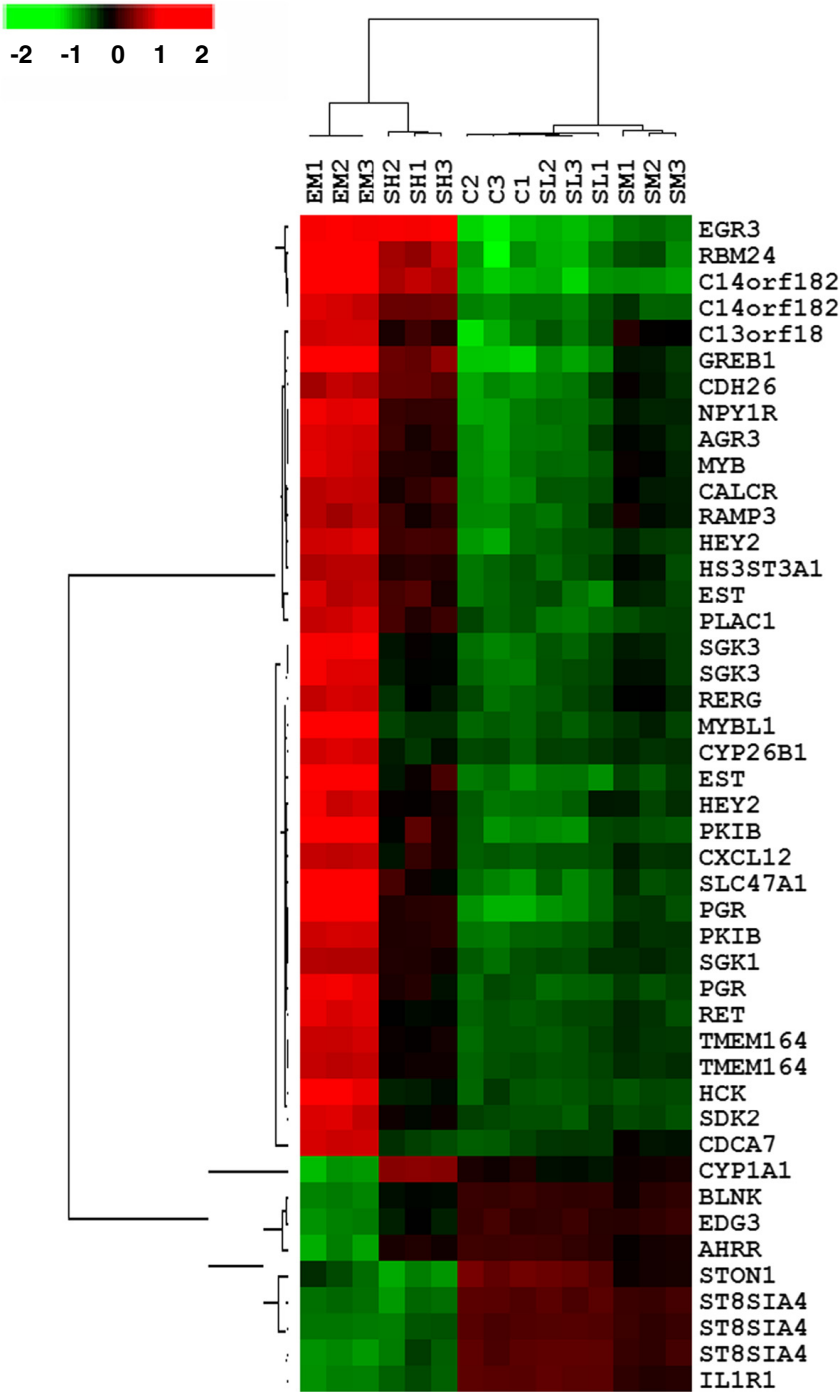
**Cluster analysis of expression profiles of 45 estrogen-responsive probes after treatment with E2 or SWT.** Gene expression profiles were obtained after treatment with 100 nM of E2 (EM), 0.0256, 0.256 and 2.56 mg/ml SWT (SL, SM and SH). The results of microarray analysis are shown as values of log_2_ fluorescent intensity. The branch lengths of the clustering tree reflect the degree of similarity of gene expression. Columns represent the gene expression levels in individual samples; rows represent individual genes. Red and green indicate transcript levels above and below the median for each gene across all samples, respectively.

We next performed hierarchical cluster analyses to group the 15 cell samples (the vehicle control group C, the E2 group EM, and the SWT groups in three concentrations, SL, SM, SH, all in triplicate) and the 45 estrogen-responsive genes on the basis of the gene expression pattern. Figure 3 shows that the profiles of MCF-7 cells treated with E2 and SH were similar but distinguishable, while both of the E2 and SH treatment groups showed dramatically different profiles compared to that of the control, SL and SM groups. The 45 genes can be clearly clustered into two gene groups, 36 E2-up-regulated probes and 9 E2-down-regulated probes. The different probes for the same genes, such as *C14orf182*, *TMEM164*, *SGK3* and *ST8SIA4*, were clustered together, further indicating the consistency in their gene expression pattern. Most E2-up-regulated genes (colored in red in the heatmap indicating increased expression) showed lower degree of up-regulation in SH treatment, except for the *EGR3* gene showing the same extent of up-regulation for both E2 and SH treatments. For a subset of genes, including *SGK3*, *RERG*, *MYBL1*, *CYP26B1*, *RET*, *HCK* and *CDCA7*, SH treatment only marginally induced the gene expression. Interestingly, E2 and SH showed the opposite effect on *CYP1A1* expression: The CYP1A1 gene was downregulated by E2 but up-regulated by SH. This difference can also be seen on the scatterplot shown in Figure 2A.

### Most SWT-responsive genes not affected by E2

**Figure 4:**
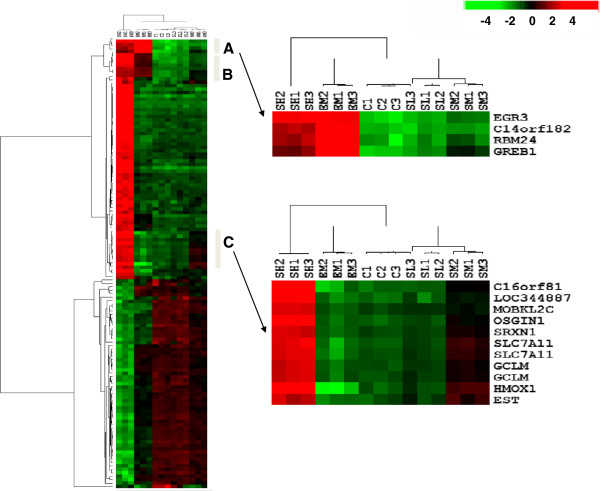
**Cluster analysis of expression profiles of 131 SWT-responsive probes after treatment with E2 or SWT.** Gene expression profiles were obtained after treatment with 100 nM of E2 (EM), 0.0256, 0.256 and 2.56 mg/ml SWT (SL, SM and SH). The results of microarray analysis are shown as values of log_2_ fluorescent intensity. The branch lengths of the clustering tree reflect the degree of similarity of gene expression. Columns represent the gene expression levels in individual samples; rows represent individual genes. Red and green indicate transcript levels above and below the median for each gene across all samples, respectively. Gene clusters denoted by the bars and letters A and B are groups containing estrogen-induced genes. Gene cluster C is the group containing genes induced by SWT in dose dependent manner but not induced by E2. The gene groups A and C are zoomed and the expression patterns with gene names are shown on the right.

The gene expression changes induced by treatment of SWT showed a dose-responsive trend, resulting changes in 1,911 unique genes (corresponding to 2,979 probe sets) from treatment with the highest concentration (SH). We applied the same criteria to identify the SWT responsive probe sets. A total of 131 probes were selected based on the filtering cutoff of fold change > 4 for up-regulated genes, fold change < 0.4 for down-regulated genes. These include 70 probes that showed strongest up-regulation and 61 probes with strongest down-regulations induced by SH treatment. We performed hierarchical clustering analyses to group the cell samples and the 131 SWT responsive genes on the basis of the gene expression pattern. Figure 4 shows that the profiles of cell samples treated with E2 and SH are obviously different. Unlike the E2 responsive genes shown in Table 1 and Figure 3, only small subset of SWT responsive genes were similarly induced by E2, including only a small group of genes in cluster A and B, which include E2-responsive genes identified in Table 1. The majority of SWT up-regulated genes were not up-reguated by E2. This may result from the high concentration used for SH treatment (2.56 mg/ml), as high concentration may induced many early response genes, which may or may not represent the pharmacological action. Therefore, genes showing dose-dependent changes after SWT treatment are particularly interesting to us. The genes in cluster C are those dose-dependently regulated by SWT, including many genes in the nuclear factor erythroid 2-related factor 2 (Nrf2) cell protective pathways, such as *HMOX1*, *GCLM* and *SLC7A11*. However, E2 treatment didn’t affect expression of these genes. This represents one of the major differences between E2 and SWT treatment.

### Microarray gene expression validated by real-time RT-PCR

**Figure 5:**
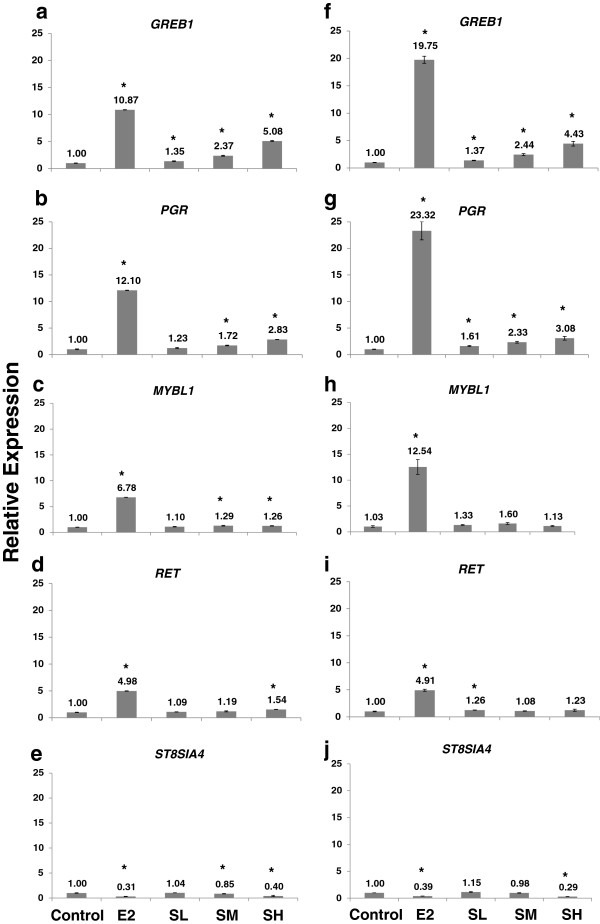
**Validation of microarray results by real-time RT-PCR.** Microarray data (**a-e**) for five genes (*GREB1*, *PGR*, *MYBL1*, *RET* and *ST8SIA4*) were compared with the results obtained by quantitative real-time RT-PCR (**f-j**). Each experiment was repeated three times and the average and S.D. are shown. *, *P* < 0.05.

The differential expression of five E2 responsive genes in response to E2 and SWT was validated by quantitative real-time RT-PCR on samples obtained from MCF-7 cells. The selected genes are E2-up-regulated genes *GREB1*, *PGR*, *MYBL1* and *RET* and E2-downregulated gene *ST8SIA4*. These genes were selected from Table 1 due to different fold change values after E2 or SWT treatment and according to their known contribution to estrogen receptor pathways known from previous studies. The fold changes of expression determined by RT-PCR for these genes were concordant with those obtained by microarrays (Figure 5). All the up-regulated genes, *GREB1*, *PGR*, *MYBL1* and *RET*, are up-regulated by E2. *GREB1* and *PGR* are up-regulated by SWT in a dose dependent manner. However, consistent with the microarray data, *MYBL1* and *RET* are induced by E2, but not by SWT.

### Dose-dependent effects of SWT and its components on growth of breast cancer cells

**Figure 6:**
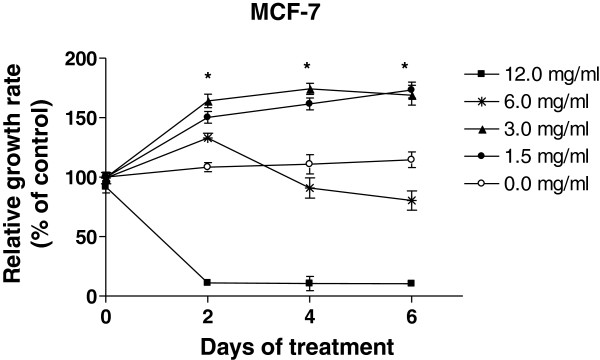
**Dose-dependent effects of SWT on MCF-7 cell proliferation.** To determine time- and dose-dependent growth rates of MCF-7 cells in the presence of SWT, cells were plated in 96-well plates (2,000 per well) in hormone-free medium for indicated times and cell growth was determined by MTS assay. Results are expressed as relative growth rate to the control cells with no drug treatment. Points, means from six replicates; bars, S.D. * p < 0.05 for all treatment groups compared to untreated control.

To examine the effects of SWT on the growth of breast cancer cells, MCF-7 cells were treated with various concentrations of SWT for 2–6 days. At low (1.5 and 3 mg/ml) concentrations, SWT stimulated MCF-7 cell growth (Figure 6); in contrast, SWT inhibited cell growth at a higher (6 and 12 mg/ml) concentration, revealing a dose-dependent activity of SWT on cell growth.

**Figure 7:**
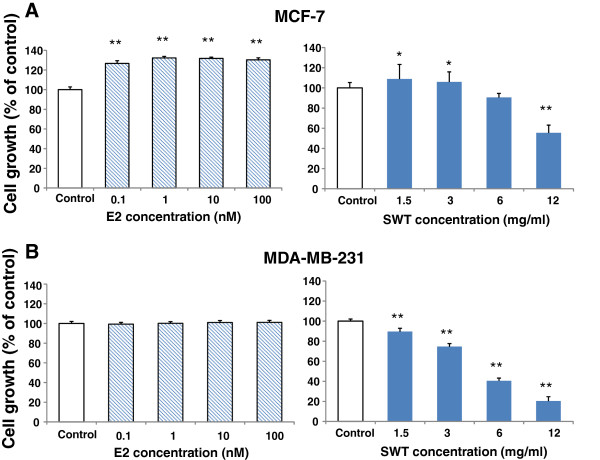
**Effects of E2 or SWT on cell growth of MCF-7 (A) and MDA-MB-231 (B).** The hormone-deprived cells in 96-well plates (5,000 cells per well) were treated with E2 or SWT for 48 hours, cell growth was measured with SRB assay. Results are expressed as percentage of control cells with no drug treatment. Points, means from three replicates; bars, S.D. *, *P* < 0.05; **, *P* < 0.01.

We next compared the effects of E2 and SWT proliferation of MCF-7 (ER+) and MDA-MB-231 (ER-) cells (48 hour treatment). Data in Figure 7A show that E2 stimulated MCF-7 cell proliferation. This effect was statistically significant at all the concentrations tested (0.1, 1, 10 and 100 nM) (*P* < 0.01). SWT showed stimulatory effect on MCF-7 cell proliferation at lower concentration (1.5 and 3 mg/ml) (*P* < 0.05), while at higher concentration (12 mg/ml) it exhibited a statistically significant cytotoxic effect (*P* < 0.01) (Figure 7A). On the contrary, neither E2 nor SWT displayed any detectable stimulatory effect on MDA-MB-231 cell proliferation (*P* > 0.05), while SWT significantly inhibited MDA-MB-231 cell proliferation in a dose dependent fashion (P < 0.01 for all the concentration tested) (Figure 7B). The cytotoxic effect of SWT on MDA-MB-231 (IC_50_ = 4.5 ± 0.21 mg/ml) was significantly stronger than that on MCF-7 cells (IC_50_ >12 mg/ml) (*P* < 0.01).

**Figure 8:**
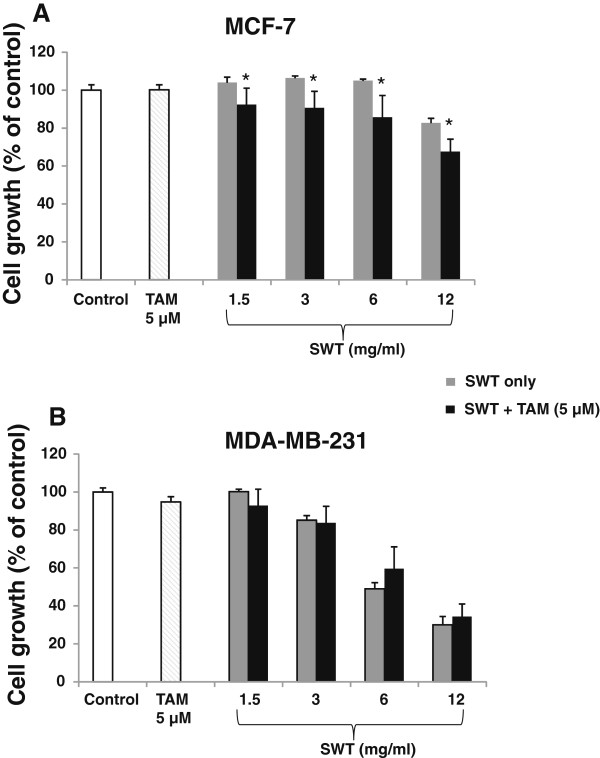
**Effects of SWT combined with tamoxifen on cell growth of MCF-7 (A) and MDA-MB-231 (B).** The hormone-deprived cells in 96-well plates (5,000 cells per well) were treated with SWT and/or tamoxifen (TAM) for 48 hours, cell growth was measured with SRB assay. Results are expressed as percentage of control cells with no drug treatment. Points, means from three replicates; bars, S.D. *, *P* < 0.05; **, *P* < 0.01.

We also assessed the combined effect of SWT and tamoxifen (TAM) on MCF-7 and MDA-MB-231 cell growth. Treatment with TAM (5 μM) alone did not affect the growth of both cell lines (Figure 8A and 8B). The growth stimulatory activity of SWT in MCF-7 cells at all concentrations was abolished by TAM treatment, indicating the ER-dependency of such activity of SWT. Co-treatment of MCF-7 cells with SWT and 5 μM TAM resulted in significantly increased inhibition of cell proliferation (*P* < 0.05) (Figure 8A). However, co-treatment of MDA-MB-231 cells with SWT combined with TAM did not result in statistically significant difference compared with SWT treatment alone (Figure 8B).

**Figure 9:**
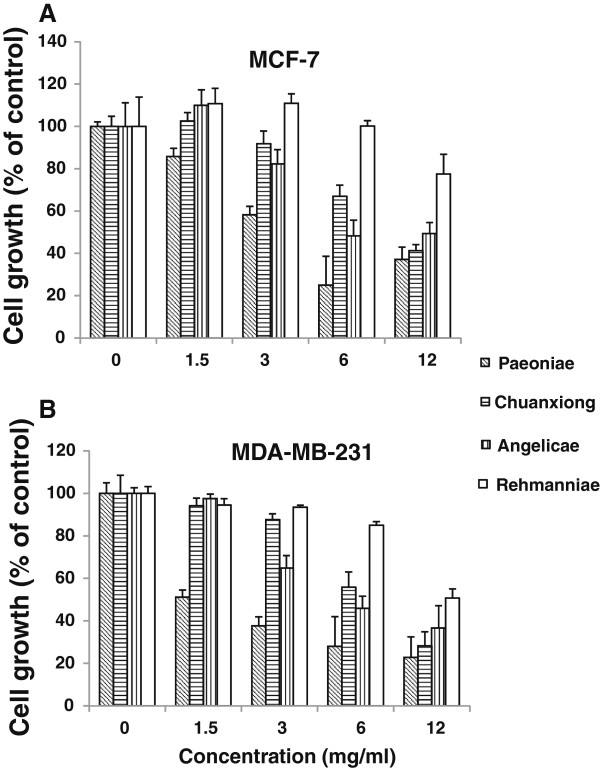
**Effects of SWT components on cell growth of MCF-7 (A) and MDA-MB-231 (B).** The hormone-deprived cells in 96-well plates (5,000 cells per well) were treated with Paeoniae, Chuangxiong, Angelicae or Rehmanniae for 48 hours, cell growth was measured with SRB assay. Results are expressed as percentage of control cells with no drug treatment. Points, means from three replicates; bars, S.D. *, *P* < 0.05; **, *P* < 0.01.

We next examined the effects of four components of SWT, Paeoniae, Angelicae, Chuanxiong and Rehmanniae, on MCF-7 and MDA-MB-231 cell proliferation. Results in Figure 9A shows Angelicae, Chuanxiong and Rehmanniae, but not Paeoniae, increased the growth of MCF-7 cells at low concentration (1.5 or 3 mg/ml), although such effect was not statistical significant (*P* > 0.05). At higher concentration, all of the components showed cytotoxicity. In MCF-7 cells, the cytotoxicity is in the order of Paeoniae (IC_50_ = 2.6 ± 0.16) > Angelicae (IC_50_ = 3.1 ± 0.073) > Chuanxiong (IC_50_ = 5.7 ± 0.57) > Rehmanniae (IC_50_ >12). In MDA-MB-231 cells, the cytotoxicity is in the same order of Paeoniae (IC_50_ = 1.1 ± 0.21) > Angelicae (IC_50_ = 2.8 ± 0.20) > Chuanxiong (IC_50_ = 5.9 ± 0.47) > Rehmanniae (IC_50_ >12) (Figure 9B).

### Dose-dependent effects of SWT and its components on estrogen receptor (ER) transcription activity

**Figure 10:**
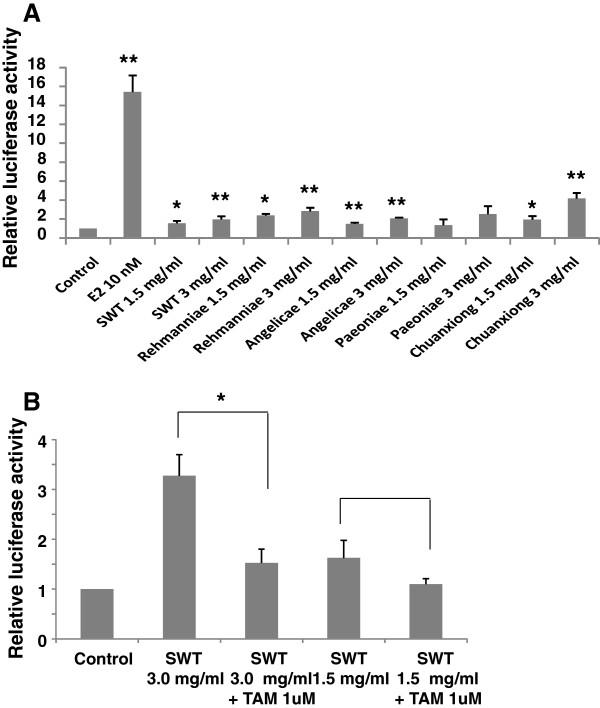
**Effect of SWT and its components on the ERE luciferase activity in MCF-7 cells.** The hormone-deprived MCF-7 cells were transiently co-transfected with the ERE-luc construct and a plasmid encoding renillar luciferase (pGL4.74) using FuGENE® HD transfection reagent. (**A**) The transfected cells were incubated in DMEM supplemented with 10% CD-FBS with E2 (10 nM), SWT or Paeoniae, Chuangxiong, Angelicae or Rehmanniae (1.5 and 3 mg/ml) for 24 h prior to measurement of firefly and renillar luciferase activities using the dual luciferase reporter gene assay. *, *P* < 0.05; **, *P* < 0.01, treatment versus control. (**B**) The transfected cells were treated with SWT 3 mg/ml or 1.5 mg/ml alone or + 1 μM tamoxifen. *, *P* < 0.05 tamoxifen + SWT versus SWT alone. Data represent the mean + SE of experiments performed in triplicate.

To confirm the estrogenic activity of SWT, we studied the effect of SWT on ER-mediated gene transcription using estrogen-responsive luciferase reporter assay. The MCF-7 cells that had been transiently co-transfected with the ERE-luciferase and a constitutively active renilla luciferase plasmid (pRL-TK-luc) were used to measure the formation of the functional ER-ERE complex in response to treatment with the E2 or SWT. Data were normalized to the renilla plasmid transfection and then expressed as a fold induction compared with untreated cells (Figure 10A). E2 (10 nM) significantly increased luciferase activity by 15 ± 1.7 fold (*P* < 0.01). SWT at concentration of 1.5 and 3.0 mg/ml had significant increased luciferase activity by 1.6 ± 0.22 and 2.0 ± 0.31 fold inductions (*P* < 0.05), respectively. All the four herbal components of SWT showed ERE activation at 1.5 and 3.0 mg/ml. The statistical significance for the ERE induction has been detected for Rehmanniae, Angelicae and Chuangxiong in dose-dependent manner (*P* < 0.05 for 1.5 mg/ml, *P* < 0.01 for 3.0 mg/ml), but not for Paeoniae (*P* > 0.05), indicating Paeoniae may not be the main component that contribute to the estrogenic activity of SWT.

We next investigated the impact of the estrogen antagonist tamoxifen on the estrogenic activity of SWT. Dual luciferase assay on MCF-7 cells was performed to determine if 1 μM TAM would inhibit the effects of SWT (1.5 and 3.0 mg/ml) in the ERE-regulated reporter. SWT at both concentrations increased ERE-transcriptional activation (Figure 10B) and such activation can be inhibited by the co-treatment of the cells with TAM (P < 0.05 for SWT 3.0 mg/ml + TAM versus SWT alone), indicating the activities may be mediated via the activation of the ER.

## Discussion

The use of traditional herbal medicine is widespread in China and other Asian countries, and is also rapidly growing in Western countries [32]. Despite its long history of use, many questions remain to be answered, due to lack of mechanistic understanding. In this study, we demonstrated, using a whole human genome microarray approach, the phytoestrogenic mechanism for a popular formula Si-Wu-Tang (SWT). Three major points are highlighted: (1) this is the first study to use DNA microarray-based gene expression analysis to reveal phytoestrogenic activity of herbal medicine. The similar approach can be used for other natural products; (2) The genomic data were validated by the “gold standard” method of gene expression study – quantitative RT-PCR; and (3) the discovery was confirmed by pharmacological assays such as cell proliferation and estrogen receptor luciferase reporter assays on breast cancer cell lines. The results presented here are very important for many women taking SWT for various conditions and clinical practitioners who recommend the use of SWT or other CAM with similar phytoestrogenic activities.

SWT has been used in China for more than 1,000 years for the relief of menstrual discomfort, climacteric syndrome, peri- or postmenopausal syndrome and other estrogen-related diseases [1-5]. The SWT formula is composed of four herbs, Radix *Rehmanniae praeparata* (Rehmanniae), Radix *Angelicae Sinensis* (Angelicae), Rhizoma *Ligustici Chuanxiong* (Chuanxiong) and Radix *Paeoniae Alba* (Paeoniae) [2]. At least nine bioactive phytochemicals have been reported for SWT: paeoniflorin, paeonol, gallic acid, ferulic acid, Z-ligustilide, ligustrazine, butylphthalide, senkyunolide A and catalpol [1]. In view of wide empiric use of SWT and known chemical components already reported, we profiled the gene expression of MCF-7 cells treated with SWT extract at the non-toxic concentration (2.56, 0.256 and 0.0256 mg/ml), its component ferulic acid (0.1, 1.0 and 10 μM) as well as β-estradiol (E2, 0.1 μM) using Affymetrix microarray HG-U133Plus2.0, enabling almost complete analysis of the transcriptome [12]. Notably, the expression of genes in the nuclear factor erythroid 2-related factor 2 (Nrf2) cytoprotective pathway were the most significantly affected by SWT, but not by β-estradiol (E2) or ferulic acid [12]. Even though the Nrf2 pathway was identified as one of the main molecular targets of SWT, it is well known that therapeutic effect of many herbal medicines can be attributed from targeting multiple rather than single molecular targets. The present study discovered that the estrogen receptor (ER) pathway represents another potential target of SWT.

Our hypothesis was derived from the “Connectivity map” (cMAP) analysis, which is based on the comparison between the database containing microarray expression data (Affymetrix HG-U133A array) from cultured cell lines (e.g., MCF-7) treated with 1,309 bioactive compounds with known mechanism of action [13] and our SWT expression data [12]. This analysis results a strongest match between the profiles of MCF-7 cells treated with SWT and those of the same cell line treated with E2 in the cMAP database [12]. Such correlation suggests an estrogenic effect of SWT. The array data for MCF-7 cells treated by E2 or SWT were further compared by correlation analysis and hierarchical clustering analysis for the similarity and difference in the treatment effects. Comparing the expression patterns using all the 54,675 probes representing all the genes in the human genome or using 45 probes selected based on fold of expression changes induced by E2, action of SWT is similar to E2 in particular when focusing on the 45-probe “estrogen-responsive genes”. The 45 probes were selected using very stringent criteria: fold change > 4 for up-regulated genes, fold change < 0.4 for down-regulated genes, and false discovery rate (FDR) < 0.01. Many of these genes have been reported to be estrogen-responsive genes *in vitro* or *in vivo*. In addition, all of the 45 probes were consistently changed in expression in E2 treated MCF-7 cells according to public available data deposited at the NCBI GEO Databases from three published studies [29-31], which used the same array type as used in our study (Affymetrix HG-U133Plus 2.0) and the cell line (MCF-7). This result indicates that the 45 probes can reliably represent the E2 regulated genes and therefore can be named as “estrogen-responsive genes” or “fingerprint of estrogen”. The correlation and clustering results revealed that the gene expression profile of MCF-7 cells for the “estrogen-responsive genes” was similarly changed by the treatment with E2 and SWT.

The list of genes includes well-known estrogen-regulated genes, such as *GREB1*, *EGR3*, *RERG*, *PGR*, and *SGK3*. Many of them can be induced by SWT. The gene for progesterone receptor, *PGR*, is an estrogen-responsive gene, whose expression has been shown to indicate a responsive estrogen receptor pathway [33]. The expression of *PGR* in MCF-7 cells or in rats can be induced by treatment with the phytoestrogens daidzein [33] and resveratrol [34], respectively. Another estrogen receptor target gene, *GREB1* (growth regulation by estrogen in breast cancer 1), is involved in the estrogen induced proliferation of breast cancer cells and has the potential of being a clinical marker for response to endocrine therapy [35]. *GREB1* can be up-regulated by several herbal medicines with phytoestronic activity, such as Chinese licorice (*Glycyrrhiza uralensis*) root [36] and the stem bark of Fabaceae (*Erythrina lysistemon*) [37] in MCF-7 cells. Our results demonstrated that SWT up-regulated the *GREB1* in dose-dependent manner. Among the 45 selected “estrogen-responsive genes”, highest up-regulation by SWT was found for *EGR3* (Early growth responsive gene 3). *EGR3* is a zinc-finger transcription factor and the bona fide target gene for ER-α [38]. Estrogen-treated MCF-7 cells showed rapid and robust induction of *EGR3*[38]. The selected 45 probes also include a few genes which have not been reported as estrogen-responsive genes before, such as *RBM24* (RNA binding motif protein 24) and *SLC47A1* (solute carrier family 47, member 1). Three probes for the *ST8SIA4* (ST8 alpha-N-acetyl-neuraminide alpha-2,8-sialyltransferase 4) consistently showed down-regulation by both E2 and SWT. The protein product of *ST8SIA4* is known to be involved in the polysialylation of neural cell adhesion molecule (NCAM), which has been linked to cancer development and dissemination [39]. *ST8SIA4* has not been reported regulated by estrogen receptors. The real-time PCR data showed a similar gene expression change of select genes in the “estrogen-responsive genes”.

Nevertheless, not all the probes of “estrogen-responsive genes” are regulated by SWT in the same way as E2. The examples include oncogenes *MYBL1*, *RET* and cyclin D1 (not listed in Table 1 due to lower fold change), which is strongly induced by E2 but not by SWT. Although the contribution of *MYBL1*, encoding for the homolog of the oncogene *MYB*, to the development of breast cancer is unknown, in a previous report it was strongly induced by E2 but only marginally by phytoestrogens such as curcumin [40]. Further study is needed to investigate the role of *MYBL1* and *RET* in estrogen induced breast cancer development. As MCF-7 (ER-positive) is a commonly used model for determining estrogenic effects, the action through ER pathways could be one of the mechanisms for SWT’s beneficial effect on alleviating postmenopausal complaints.

The gene expression profiles for SWT and E2 also showed a strong difference. A wider range of cellular pathways and targets were affected by SWT but not E2. Hence, the action of SWT on MCF-7 cells is multifaceted. One of the most notable differences is the ability to induce the Nrf2. Although Nrf2-mediated oxidative stress response was identified as the pathway most significantly changed among differentially expressed genes showing dose-dependent response to SWT treatment, this trend has not been observed for E2 treatment. This finding suggests that SWT could have cancer preventive effect. The role of estrogen in the initiation and progression of breast cancer has been well known [41]. However, there is a large body of evidence that the consumption of phytoestrogens derived from natural products can decrease the risk of cancer although they display estrogen-like activity [42]. These results support a notion that SWT may not have the cancer-causing effects of estradiol, but have the beneficial cell protective activity.

To confirm the phytoestrogenic action of SWT, we examined the effect of SWT alone or in combination with tamoxifen, on the growth of estrogen-dependent MCF-7 cells and estrogen-independent MDA-MB-231 breast cancer cell lines. Firstly we found that SWT, similar to E2, can stimulate the proliferation of MCF-7 cells, but not MDA-MB-231 cells. Such effect is dose-dependent. At low concentrations, SWT stimulated cell growth, while at high concentrations, SWT showed cytotoxicity. On the MDA-MB-231 cells, SWT failed to show any growth stimulating effect, but has stronger cytotoxic effect than MCF-7 cells. Thus, the growth stimulating effect may be mediated by the ER, while the cytotoxic effect of SWT on both MCF-7 and MDA-MB-231 cells may involve estrogen receptor-independent pathways. These results are in agreement with those of Chang et al. (2006) who reported SWT and its constituent ferulic acid caused MCF-7 cell proliferation [25,43]. While in general SWT have relative safe record in clinical usage, potential harmful effects may exist for patients with breast cancer. In particular for ER-positive breast cancer, use of SWT may promote the tumor cell growth and counteract the effects of estrogen-deprivation treatment by tamoxifen or aromatase inhibitors. Similar issues have been raised for other phytoestrogens [14].

The growth inducing effect can be attenuated by the treatment with tamoxifen, an antagonist of the estrogen receptor, further indicating such effect may be ER-dependent. Tamoxifen inhibits E2-mediated effects by competing for receptor binding [44]. Although tamoxifen alone did not affect the growth of MCF-7 and MDA-MB-231 cells, co-treatment of SWT and tamoxifen resulted in a dose-dependent decrease in cell growth. Such combined effect was significant for SWT concentration as low as 1.5 mg/ml. This effect was not significant in the MDA-MB-231 cells. Thus, this combined growth inhibitory effect may be mediated by estrogen receptor dependent mechanism. Over the last decade, breast cancer prevention has focused mainly on endocrine therapies using selective estrogen receptor modulators such as tamoxifen. The use of tamoxifen is able to reduce incidence of ER-positive cancer in high-risk women [44]. However, tamoxifen have not been widely adopted as a preventive strategy for long-term use, due to lack of complete prevention as well as intolerable side effects, including endometrial cancer, thromboembolic events and liver cancer [45]. Moreover, tamoxifen have no effect in reducing the risk of ER-negative tumors. As breast cancer remains a global public health challenge, there is a need for developing effective and non-toxic preventive agents. The chemoprevention effectiveness of SWT alone or in combination with tamoxifen needs further evaluation.

Because phytoestrogens structurally resemble estrogen, these compounds may exert their effects primarily through binding to ER, although usually with a weaker affinity than endogenous estrogens [46]. There are two types of ER, alpha (ER-α) and beta (ER-β), both mediating the action of physiological endogenous estrogens (for reviews, see [46] and [47]). ER-α and ER-β differ in their functions and tissue distributions. Studies in MCF-7 breast cancer cells showed that ER-α exerts a proliferative effect while ER-β is not necessary for proliferation but against the effects of ER-α [48]. Many phytoestrogens, including resveratrol, genistein, and daidzein, have been shown able to bind both ER-α and ER-β and to modulate the transcription of estrogen-responsive target genes in a dose-dependent manner [48]. The four herbal components of SWT were studied for their possible estrogen-like activities using cell growth assay and ERE luciferase assay. Our results indicate that Rehmanniae, Angelicae and Chuangxiong are more likely contribute to the overall phytoestrogenic activity of SWT. Paeoniae is less likely to play a main role for the estrogenic effect because it did not show significant effect on growth induction and ERE activation in MCF-7 cells. However, the cytotoxicity activity of Paeoniae on both MCF-7 and MDA-MB-231 cells is the most potent among all the herbal components. This result suggests that Paeoniae may have a potential anticancer activity on both ER + and ER- breast cancers. In a recently published work [49], 38 compounds from SWT series were studied for the binding to the ER-α using a stably-transfected human breast cancer cell line MVLN. Among all the compounds tested, 22 compounds, including organic acids and flavones, showed estrogen-like activity at the concentration higher than 20 μg/ml. These results indicate that the overall estrogen-like activity of SWT is attributed to multiple components and compounds. The synergistic or antagonistic interactions of these components remain to be studied. Further studies also need to obtain the information for SWT components on the specificity and selectivity of targeting the ERs. Because activating ER-β may prevent breast cancer [50], such results will be essential for the evaluation of SWT as a cancer preventive agent.

## Conclusions

Although SWT is a widely used oriental medicinal formula, the scientific evidence to prove its efficacy or side effects remains insufficient. In this study, gene expression profiles obtained by genomic approach based on DNA microarray analysis shed light on the new molecular mechanism of SWT. The identified novel phytoestrogenic activity of SWT supports its current use for alleviating postmenopausal conditions and possibly for breast or prostate cancer prevention. Since carcinogenesis involves multiple abnormal genes/pathways, using herbal medicines in cancer prevention may be superior to the agents targeting a single molecular target. The application of SWT, due to of its low cost and low toxicity, may have a profound impact on human health. Further work is needed to determine the *in vivo* relevance of the *in vitro* findings obtained from the present study. The approach used in this study, genomic analysis following by functional validation, proved to be powerful in an understanding of mechanisms of actions for CAM as exemplified by our study with SWT. There is a potential to apply this approach for many other CAM and natural products. Furthermore, the gene expression changes identified in this study could be used as biomarkers for assessing the intact quality of SWT or its series decoctions including Xiang-Fu-Si-Wu decoction, Tao-Hong-Si-Wu decoction, Qin-Lian-Si-Wu decoction, and Shao-Fu-Zhu-Yu decoction. The genomic approach can be integrated with traditional chromatography-based fingerprinting method, metabolomics, and pharmacological assays to obtain a complete understanding of herbal medicines.

## Abbreviations

SWT: Si-Wu-Tang; E2: β-estradiol; ERE: Estrogen-responsive element; ER: Estrogen receptor; CAM: Complementary and alternative medicine; cMAP: Connectivity Map; FBS: Fetal bovine serum; CD-FBS: Charcoal-dextrin stripped FBS; GEO: Gene Expression Omnibus; SRB: Sulforhodamine B; Nrf2: Nuclear factor erythroid 2-related factor 2.

## Competing interests

The authors declare that they have no competing interests.

## Authors’ contributions

ML carried out the real-time PCR analysis, most of the pharmacology studies, and participated in the data analysis. JF and SW performed the cell proliferation and luciferase assays. ZW helped with the preparation of samples. ZW, CW, LS and ZW carried out the microarray analysis. ZZ and MC participated in the design of the study. YH and MC conceived of the study, and participated in its design and coordination and drafted the manuscript. All authors read and approved the final manuscript.

## Pre-publication history

The pre-publication history for this paper can be accessed here:

http://www.biomedcentral.com/1472-6882/13/11/prepub
